# A clinical evaluation of HyperSight CBCT enhanced offline adaptive breast radiotherapy

**DOI:** 10.3389/fonc.2026.1912819

**Published:** 2026-08-19

**Authors:** Jina Kim, Mi-Hwa Kim, Hae-Jin Park

**Affiliations:** Department of Radiation Oncology, Ajou University School of Medicine, Suwon, Republic of Korea

**Keywords:** breast cancer, CBCT, Halcyon, HyperSight, image quality, offline adaptive radiotherapy

## Abstract

**Introduction:**

Anatomical changes such as seroma shrinkage, soft-tissue deformation, and tumor-bed displacement frequently occur during breast radiotherapy and may compromise target coverage or increase unnecessary dose to surrounding tissues when the initial plan is maintained throughout treatment. Offline adaptive radiotherapy (ART) requires CBCT systems with CT-equivalent HU accuracy and robust dose-recalculation accuracy to reliably quantify these changes. This study evaluated whether the newly integrated HyperSight CBCT on the Halcyon platform provides high image quality, HU-to-electron density accuracy, and dosimetric reliability to support offline ART in breast cancer.

**Methods:**

Twelve patients who underwent treatment with both Halcyon 3.1 and Halcyon-HyperSight systems were analyzed. Image quality was assessed using structural similarity metrics (SSIM, RMSE, NRMSE), uniformity, signal and noise characteristics, contrast-to-noise ratio (CNR), and edge-based indices. HU-to-ED curves were generated using a tissue-equivalent electron-density phantom and applied for CBCT-based dose recalculation.

**Results:**

Compared with Halcyon 3.1, HyperSight demonstrated significantly improved structural similarity to simulation CT, with higher SSIM and lower RMSE/NRMSE, as well as significantly improved uniformity and edge sharpness. HU-to-ED linearity for HyperSight closely matched simulation CT across soft-tissue and bone-equivalent materials, whereas Halcyon 3.1 exhibited HU bias and large variability. Dosimetrically, HyperSight accurately reproduced CT-based CI_Paddick and HI values, showing markedly reduced deviations relative to Halcyon 3.1. Clinical evaluation further showed that HyperSight identified volumetric and positional changes in both the whole breast and tumor bed, identifying regions susceptible to high-dose spill or under-coverage when no plan adaptation was performed.

**Discussion:**

These findings suggest that HyperSight provides HU accuracy closer to that of simulation CT, improved image quality, and more accurate CBCT-based dose recalculation than Halcyon 3.1, thereby supporting the identification of clinically meaningful anatomical changes. HyperSight may therefore serve as a practical imaging option for offline ART in breast radiotherapy and may help reduce reliance on repeat simulation CT in selected cases while supporting adaptive treatment assessment.

## 1 Introduction

Breast cancer remains one of the most common malignancies among women worldwide, and adjuvant radiotherapy following breast-conserving surgery is a well-established standard of care that reduces local recurrence and improves survival by delivering adequate doses to the tumor bed and surrounding tissues (1, 2). Seroma, frequently observed after surgery, gradually resolves or changes in shape during treatment. These changes can shift the tumor-bed position and reduce the target volume (3, 4). When the initial treatment plan is maintained despite such anatomical changes, an unnecessary dose may be delivered to normal tissues while target coverage is compromised, ultimately reducing therapeutic effectiveness and increasing the risk of toxicity (5, 6). These dynamic anatomical variations highlight the need for periodic evaluation and adjustment of the treatment plan, underscoring the emerging clinical demand for adaptive radiation therapy (ART).

ART compensates for patient-specific anatomical changes by incorporating repeated imaging and either online or offline plan adaptation, enabling more precise and effective radiation delivery. Offline ART, in particular, updates the treatment plan before subsequent fractions based on the daily verification images acquired for setup correction, and is therefore more practical for breast cancer, where anatomical changes accumulate gradually rather than abruptly (7–9). Because seroma shrinkage and soft-tissue deformation often progress throughout the treatment course, offline ART may provide an optimal balance between clinical feasibility and resource efficiency. By mitigating unintended dose to normal tissues and improving target conformity, offline ART has the potential to enhance individualized treatment quality in breast radiotherapy. Although anatomical changes during breast radiotherapy, particularly seroma shrinkage, are well documented, most previous studies have been limited by insufficient CBCT HU accuracy, making dose-recalculation for offline ART impractical. Therefore, a system capable of CT-equivalent HU accuracy is essential to translate breast ART into routine clinical practice.

The newly introduced Varian Halcyon-HyperSight CBCT (hereafter referred to as HyperSight) provides a 6-second acquisition time, an extended field of view (up to 70 cm), an enhanced iterative reconstruction algorithm (iCBCT-Acuros), and metal-artifact reduction (MAR), thereby reducing motion blur, truncation artifacts, and scatter effects while improving image quality and HU consistency under low volume computed tomography dose index (CTDI_vol) conditions (10–12). The larger FOV and rapid acquisition are advantageous for imaging the entire breast, chest wall, and regional lymphatics, while improved HU accuracy directly influences the reliability of HU-to-electron density (ED) conversion for dose recalculation. These features are particularly important for offline ART workloads, which rely on accurate CBCT imaging to update treatment plans without requiring repeat simulation CT. Such improvements may reduce patient time in the department, avoid unnecessary imaging dose, and enhance the overall efficiency and comfort of radiotherapy.

By contrast, the previous Halcyon 3.1 CBCT (hereafter referred to as Halcyon 3.1) had several limitations: (1) restricted FOV resulting in truncation and loss of peripheral structures, (2) longer acquisition times with increased susceptibility to respiratory or positional motion, (3) limited MAR and calibration performance, and (4) degraded HU accuracy, collectively undermining target/OAR delineation and HU-based dose recalculation (13, 14). Therefore, before adopting HyperSight for offline ART in breast radiotherapy, it is essential to systematically evaluate its image quality, HU-to-ED accuracy, and the extent to which CBCT-based dose recalculation can reproduce CT-based plan quality.

This study aimed to evaluate whether HyperSight can serve as a reliable imaging platform for offline ART in breast cancer, potentially reducing the need for repeat simulation CT. Specifically, we (i) compared HyperSight and Halcyon 3.1 image quality against simulation CT, (ii) validated HU-to-ED curves using an electron density phantom, and (iii) assessed whether CBCT-based dose recalculation using HyperSight could reproduce simulation CT–based conformity and homogeneity with the same beam configurations. Through this investigation, we sought to determine whether HyperSight can support clinically acceptable plan adaptation without re-simulation and to explore its potential to improve workflow efficiency in a clinical environment where routine re-CT simulations for all patients may be impractical.

## 2 Materials and methods

### 2.1 Patient selection

This study included breast cancer patients who underwent treatment using both the Halcyon 3.1 and Halcyon-HyperSight systems, allowing direct comparison of images acquired on different platforms within the same individual. A total of 12 patients were analyzed, comprising seven right-breast and five left-breast cases. All patients had undergone breast-conserving surgery, and the breast anatomy was preserved because none had undergone total mastectomy or breast reconstruction. The treatment volume was limited to the whole breast and did not include regional lymph nodes, including the axillary nodes. Consequently, glandular breast tissue remained clearly visible outside the postoperative tumor bed, and no reconstruction-related metallic or silicone implants were present. Surgical clips placed during surgery were retained and served as anatomical landmarks for tumor-bed localization. All patients received whole-breast irradiation with a prescription of 40 Gy in 16 fractions, followed by a tumor-bed boost of 10 Gy in 4 fractions. For image comparison, clinically acquired Halcyon 3.1 scans and HyperSight scans obtained during routine treatment were used. To minimize temporal anatomical variability, only image sets acquired within ±1 day between the two systems were included.

This investigation was conducted as a retrospective analysis of images generated during standard clinical care, without any influence on patient management or treatment delivery. The study was approved by the Institutional Review Board of Ajou University Hospital (IRB No. AJOUIRB-DB-2024-483), which waived the requirement for informed consent given the retrospective nature of the study, and all patient data were fully de-identified prior to analysis.

### 2.2 Image acquisition

Simulation CT scans used as the reference for image‐quality and dosimetric evaluation were acquired on the Aquilion Exceed LB system (Canon Medical Systems, Japan) using a breast protocol (120 kVp, automatic mAs). The average CTDI_vol was 7.53 mGy, and the slice thickness was 5 mm. Both the data collection diameter and reconstruction diameter were set to 700 mm.

Halcyon 3.1 CBCT images were acquired on the Halcyon 3.1 platform (Varian Medical Systems, USA) using the breast protocol (125 kVp, 300 mAs) with the iCBCT reconstruction algorithm. The average CTDI_vol was 6.04 mGy with a slice thickness of 2 mm. HyperSight CBCT images were obtained on the Halcyon 4.0 system using the breast protocol (125 kVp, 175 mAs) with the enhanced iCBCT-Acuros reconstruction algorithm, yielding an average CTDI_vol of 3.39 mGy and a 2-mm slice thickness. All CT and CBCT images for all patients were acquired under free-breathing conditions.

HyperSight incorporates substantial hardware and software improvements over Halcyon 3.1 (15, 16). Hardware upgrades include a more advanced anti-scatter grid, a new HU calibration phantom containing solid-water inserts, multi-kVp HU calibration, and hardware-based scatter correction, collectively enhancing HU accuracy and image uniformity. Software advances include a statistical iterative reconstruction algorithm with integrated Acuros CT scatter reduction, gradient-weighted motion-resilient reconstruction to mitigate motion artifacts near the body surface, and a metal artifact reduction (MAR) module. Additionally, gantry rotation speed was increased to 6 RPM, reducing scan time to approximately 6 seconds, and the field of view expanded from 491 mm to up to 700 mm. These system-specific characteristics were recorded, and acquisition protocols were standardized across modalities to ensure fair comparison of image-quality and quantitative metrics.

### 2.3 Image registration and evaluation

#### 2.3.1 Image registration

**Figure 1:**
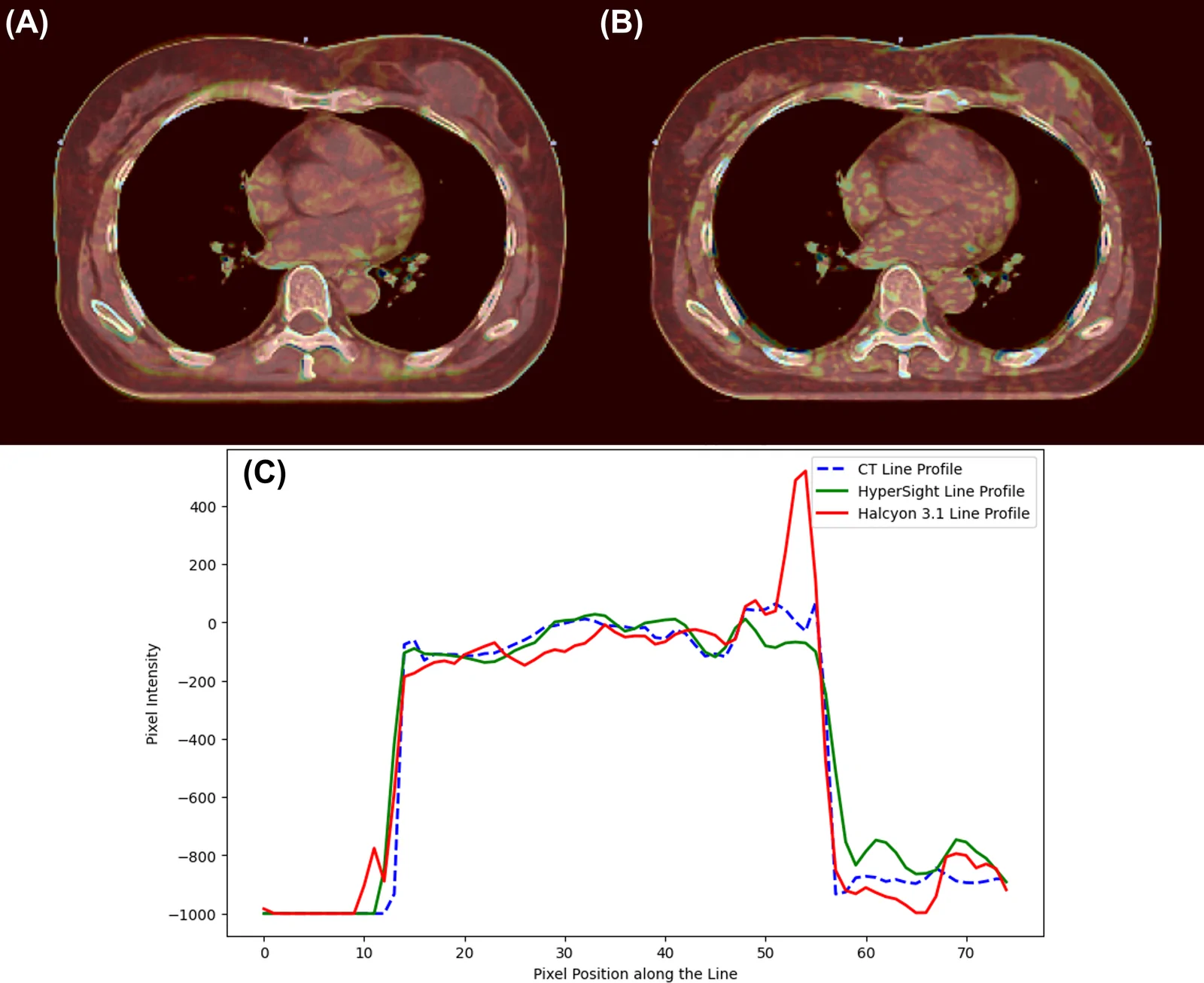
Image registration: **(A)** simulation CT–HyperSight, **(B)** simulation CT–Halcyon 3.1, **(C)** pixel-to-pixel matched line profiles.

All image registrations were performed using in-house software developed in Python (pydicom, NumPy, and SciPy). As shown in **Figures 1A, B**, all images were rigidly registered to the simulation CT. The CT slice for registration was selected based on the consistent visualization of well-defined anatomical landmarks across all three modalities, including the glandular tissue, adjacent ribs, and the lung–chest wall boundary. The corresponding slices in the HyperSight and Halcyon 3.1 datasets were identified using these same landmarks to ensure consistent slice selection. Because the pixel spacing and image matrix differed slightly among the three modalities, each selected image was resized to a common image size. The number of image channels was standardized, and padding was applied where necessary to ensure identical image dimensions before pixel-wise analysis. A global rigid registration (translation and rotation) was performed manually by an experienced medical physicist. Rotational adjustments were limited to ≤0.3° to reflect the clinical constraint that Halcyon 3.1 does not support 6-degree-of-freedom couch corrections. Rigid registration was used rather than deformable registration, as deformable algorithms can introduce algorithm-dependent structural warping and pixel displacement, thereby preventing reliable pixel-to-pixel and anatomy-preserving comparisons across modalities.

Following the initial global alignment, a region of interest (ROI) encompassing well-defined glandular tissue was manually placed at the same anatomical location in all three modalities for local refinement. The ROIs were matched to one another based on the internal glandular tissue pattern to optimize local anatomical correspondence. Within this ROI, pixel position–intensity line profiles were extracted in the x- and y-directions (**Figure 1C**), and line-shift analysis of corresponding profile features was used to quantitatively measure and correct the residual misalignment among the simulation CT, HyperSight, and Halcyon 3.1 datasets. After refinement, the residual shift was within 3 pixels in both directions for all datasets (pixel spacings: 0.977, 1.051, and 0.959 mm for the simulation CT, HyperSight, and Halcyon 3.1, respectively). The registration-refined datasets were then used for all quantitative image-quality assessments.

#### 2.3.2 Image evaluation

Image quality was quantified using the following metrics: SSIM, RMSE, NRMSE, image uniformity, signal, noise, SNR, contrast, CNR, and edge-based indices (EPI and EPS).

##### 2.3.2.1 Structural similarity metrics (SSIM, RMSE, NRMSE)

SSIM was calculated within a 50×50 mm ROI to assess structural similarity to the simulation CT, incorporating luminance, contrast, and structural components, as shown in Equation 1 (17). Values closer to 1 indicate higher similarity to the reference CT.

(1) $$SSIM\;=\;l{\left(x,\;y\right)}^{\alpha }\;c{\left(x,\;y\right)}^{\beta }\;s{\left(x,\;y\right)}^{\gamma }$$

RMSE was computed to quantify pixel-wise intensity deviations from the simulation CT, and NRMSE was derived by normalizing RMSE to account for differences in image dynamic range (18).

##### 2.3.2.2 Uniformity metrics

Uniformity was evaluated using four peripheral ROIs (10×10 mm each), spaced ≥50 mm apart within homogeneous adipose tissue. Central ROI placement was not feasible due to heterogeneous thoracic anatomy, consistent with prior clinical CT/CBCT studies in the breast and chest (19). Mean HU and standard deviation within each ROI were used to quantify uniformity.

##### 2.3.2.3 Signal and contrast metrics (signal, SNR, contrast, CNR)

Signal was defined as the mean HU within a 10 × 10 mm ROI placed in glandular tissue, while noise was derived from the standard deviation of HU measured in an adipose-tissue ROI of the same size. SNR was calculated as the ratio of signal to noise, representing the stability of tissue signal relative to image noise (20).

Contrast was measured as the HU difference between glandular tissue and adipose tissue. CNR was obtained by normalizing this contrast to the noise level, reflecting the ability to distinguish adjacent soft-tissue structures.

##### 2.3.2.4 Edge-based metrics (EPI, EPS)

Edge sharpness was assessed using edge-profile analysis adapted from the edge-response approach described by Fujita et al. (21). A 10 × 10 mm ROI was positioned across a tissue boundary to evaluate edge characteristics.

EPI (Edge Profile Intensity) was used to quantify the intensity transition across the selected tissue boundary, providing an index of edge definition.

EPS (Edge Profile Smoothness) was defined as the standard deviation of intensity values within the selected edge ROI. Lower EPS values indicate greater edge smoothing (i.e., increased edge blurring), whereas higher EPS values indicate better preservation of edge-intensity transitions.

### 2.4 Treatment planning and dosimetric evaluation

#### 2.4.1 Electron-density curve generation

**Figure 2:**
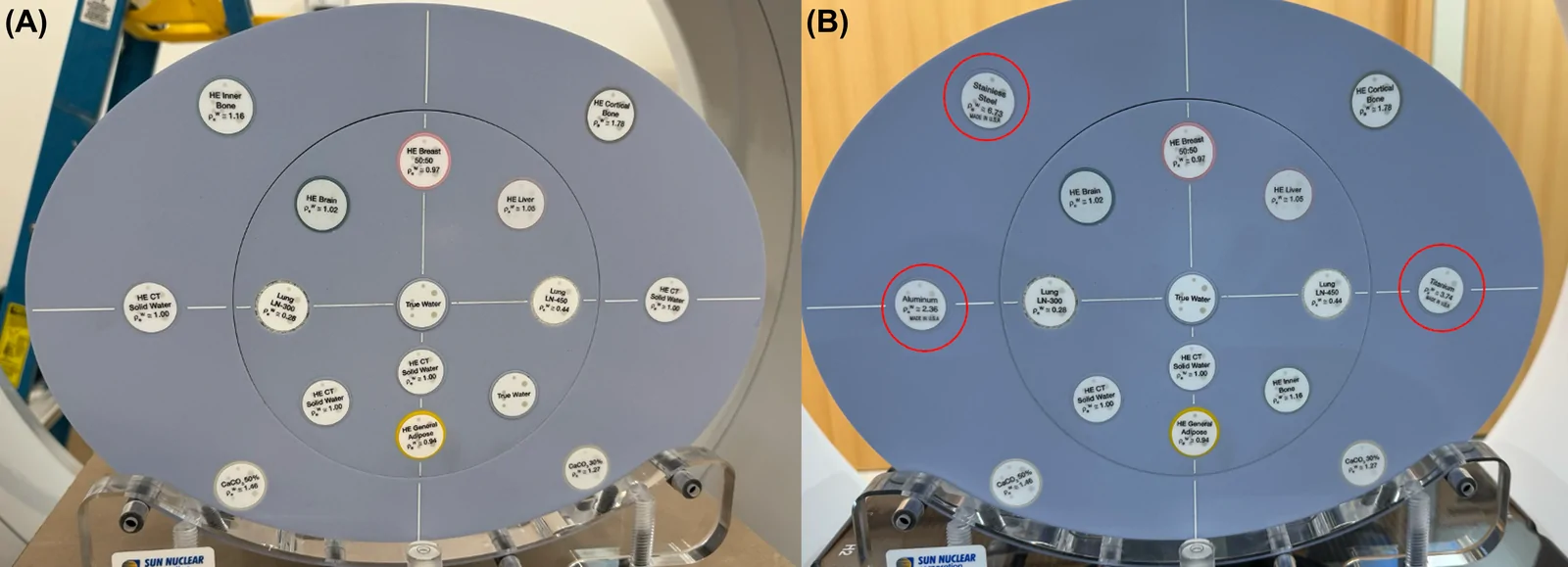
Phantom and plugs used for electron density measurement: **(A)** insertion of non-metal plugs only, and **(B)** insertion of both non-metal and metal plugs (metal plug indicated by red circle).

To assess the accuracy of CBCT-based dose recalculation for offline adaptive radiotherapy, HU-to-ED curves were independently generated for the simulation CT, HyperSight, and Halcyon 3.1 systems. Measurements were performed using the CBCT Electron Density Phantom (Model 062MA, Sun Nuclear, USA) under identical positioning and acquisition conditions (120–125 kVp). Two scanning configurations were evaluated:

1. a setup containing 16 non-metallic tissue-equivalent inserts (**Figure 2A**), and
2. a mixed setup including 13 non-metallic inserts and 3 metallic inserts (aluminum, titanium, stainless steel) (**Figure 2B**).

Insert positions and orientations were kept identical across all imaging modalities to ensure direct comparability.

The non-metallic inserts represented ICRU-44/ICRP-110–based reference tissues, including lung (LN-300, LN-450), adipose, breast 50/50, true water, solid water, brain, liver, inner bone, and CaCO_3_-based trabecular/cortical bone materials. Metallic inserts were composed of aluminum, titanium, and stainless steel (22, 23). To verify system calibration, two true-water plugs (1.00 g/cm³) and four solid-water plugs (1.021 g/cm³) were included, and their measured HU values were required to fall within the accepted water tolerance range (0 ± 10 HU). This step ensured the reliability of HU-to-ED conversion for each imaging system.

All image sets were processed using RapidCHECK v3.0.1 (Sun Nuclear, USA) to generate HU-to-ED conversion curves. These modality-specific ED curves were subsequently imported into the Eclipse v18 Treatment Planning System (Varian Medical Systems, USA) and applied directly for CBCT-based dose recalculation.

#### 2.4.2 Treatment planning

All treatment plans were generated according to the institutional breast radiotherapy protocol. Organs at risk (OARs) were first contoured using an AI-based auto-segmentation system (AccuContour v3.1, Manteia, China), after which all structures were reviewed and, when necessary, corrected by an experienced radiation oncologist to ensure clinical accuracy. Treatment planning was performed in Eclipse v18 (Varian Medical Systems, USA) using a VMAT technique with the Acuros XB (AXB) dose calculation algorithm. All plans employed 6-MV flattening-filter-free (FFF) beams at a dose rate of 800 MU/min. For each patient, three to four arcs were employed, and beam geometry was optimized according to the laterality of the treated breast. In left-sided cases, gantry angles were arranged approximately from 160° to 300°, whereas right-sided treatments used arcs spanning 200° to 60°. Collimator rotations between 5° and 10° were applied to mitigate tongue-and-groove effects and improve fluence modulation. These configurations were selected to ensure adequate PTV coverage while minimizing dose to adjacent organs at risk.

##### 2.4.2.1 CBCT-based dose recalculation

For dose recalculation, HyperSight and Halcyon 3.1 CBCT images were rigidly registered to the simulation CT. The original fluence map and all beam parameters were then transferred to each CBCT dataset. During recalculation, the corresponding modality-specific ED curve was applied to ensure accurate HU-to-ED conversion for each CBCT system.

##### 2.4.2.2 Adaptive planning and treatment

Offline adaptive radiotherapy was considered when routine CBCT images demonstrated clinically meaningful anatomical changes, including changes in target volume and/or position relative to the original treatment plan. The decision to perform adaptive treatment was made by the treating radiation oncologist based on clinical judgment. Of the 12 adaptive plans, 11 involved replanning of the tumor-bed boost, whereas one involved replanning of the whole-breast treatment.

For adaptive planning, organs at risk (OARs) were initially generated using an AI-based auto-segmentation system and reviewed and edited as necessary. The target volume was redefined by the treating radiation oncologist through comparison with the simulation CT, using surgical clips and the updated CBCT anatomy as anatomical references. Because anatomical changes modified the target volume and/or its position, treatment plans were re-optimized and recalculated using the updated contours. Patient-specific delivery quality assurance (DQA) was performed before treatment, and the adapted plans were subsequently used for clinical patient treatment.

To quantitatively evaluate anatomical changes, the spatial mismatch between the initial and adaptive PTVs was analyzed. The hot region (i.e., the overdose volume) was defined as the volume included in the initial PTV but excluded from the adaptive PTV (initial PTV − adaptive PTV), representing tissue that would continue to receive unnecessary prescription dose if the original treatment plan were maintained. Conversely, the cold region (i.e., the underdose volume) was defined as the volume included in the adaptive PTV but absent from the initial PTV (adaptive PTV − initial PTV), representing potential target under-coverage.

#### 2.4.3 Dosimetric evaluation

Plan quality was assessed using the Paddick Conformity Index (CI_Paddick) and the Homogeneity Index (HI). The Paddick conformity index was selected because it evaluates both target coverage and unnecessary irradiation outside the target volume, providing a more comprehensive assessment of dose conformity. CI_Paddick quantifies the precision with which the prescription isodose conforms to the PTV, with values approaching 1 indicating superior conformity (24). HI characterizes the uniformity of dose distribution within the PTV, where lower values indicate greater homogeneity (25). These indices were compared across simulation CT, HyperSight, and Halcyon 3.1 dose-recalculated plans to evaluate the extent to which each CBCT modality reproduced CT-based dosimetric performance. In addition, DVH-based dosimetric parameters were compared between the simulation CT and each CBCT-based dose recalculation. PTV evaluation included Dmean, D95, D98, D2, D50, V95%Rx (the percentage of the PTV receiving at least 95% of the prescription dose), and V107%Rx (the percentage of the PTV receiving at least 107% of the prescription dose) to assess target coverage and dose distribution. Organ-at-risk evaluation included the ipsilateral lung (Dmean, V5Gy, V10Gy, and V20Gy), heart (Dmean and Dmax), contralateral breast (Dmean, Dmax, and V5Gy), and global maximum dose.

### 2.5 Statistical analysis

All quantitative metrics were analyzed on a per-patient paired basis, allowing direct comparison between imaging modalities. Normality of the data distribution was assessed using the Shapiro–Wilk test. For metrics that met normality assumptions, paired t-tests were performed; otherwise, comparisons were conducted using the Wilcoxon signed-rank test. To evaluate the relative performance of the three imaging modalities (simulation CT, HyperSight, and Halcyon 3.1), mean values across modalities were compared for each imaging and dosimetric parameter. Effect sizes were additionally calculated using Cohen’s dz for paired t-tests and rank-biserial correlation for Wilcoxon signed-rank tests. Ninety-five percent confidence intervals (95% CIs) were also calculated for paired mean differences to provide estimates of the magnitude and precision of the observed differences. All statistical analyses were conducted in Python 3.11, using pandas and NumPy for data handling and SciPy (scipy.stats) for hypothesis testing. A two-tailed significance threshold of p < 0.05 was adopted for all comparisons.

## 3 Results

### 3.1 Structural similarity metrics (SSIM, RMSE, NRMSE)

**Figure 3:**
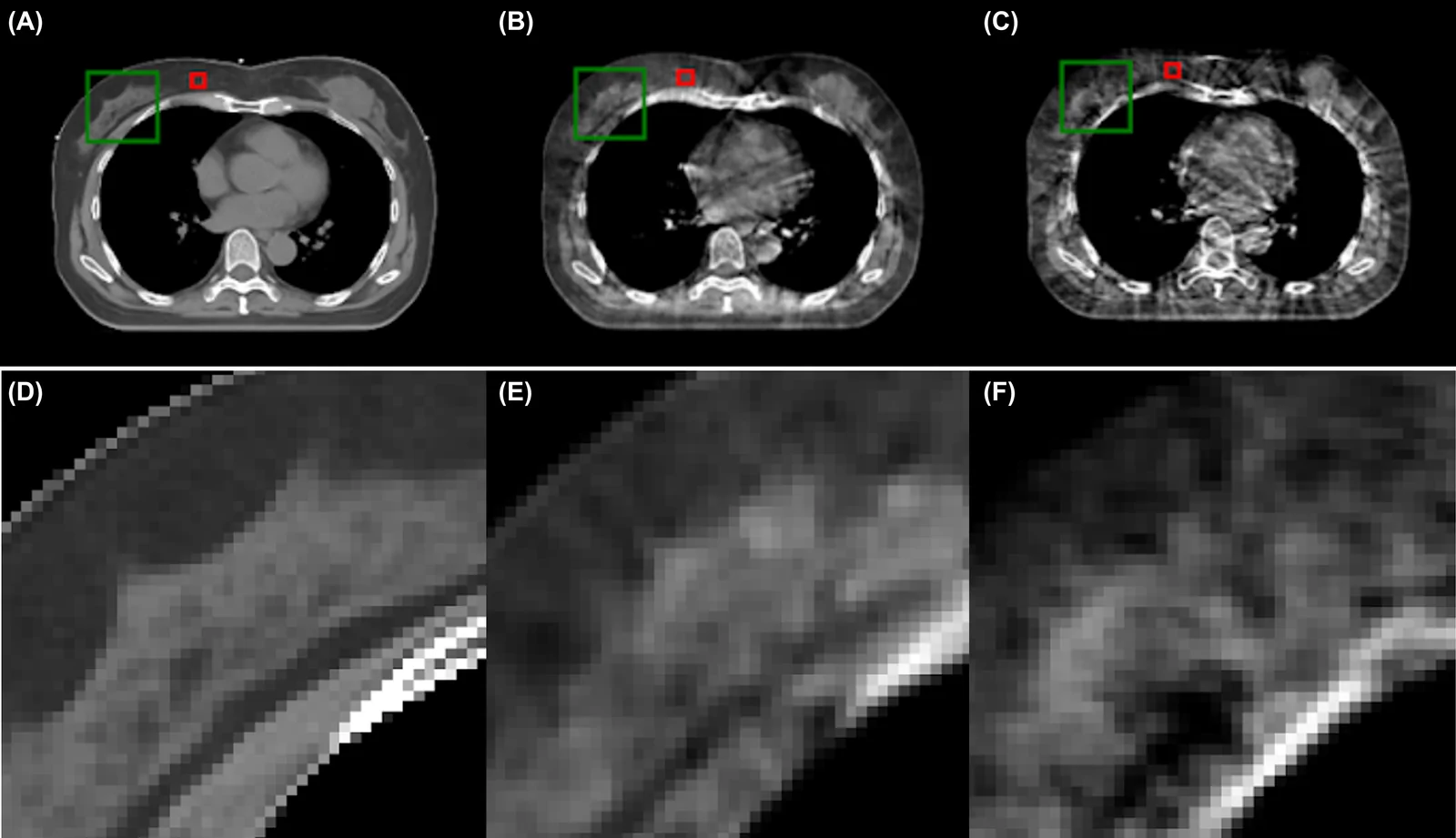
Comparison of aligned images and ROI regions for Simulation CT, HyperSight, and Halcyon 3.1. **(A–C)** Original images for each modality, and **(D–F)** magnified ROI images corresponding to the green box. The green box (50 × 50 mm) was used for SSIM calculation, and the red box (10 × 10 mm) was used for RMSE calculation.

**Figure 3** illustrates representative slices from the three modalities after registration to the simulation CT. Halcyon 3.1 images consistently exhibited more pronounced artifacts compared with HyperSight, with visibly blurred glandular boundaries and reduced delineation of fine parenchymal structures in the magnified ROI. In contrast, HyperSight demonstrated more homogeneous soft-tissue contrast and sharper structural definition, resulting in closer visual agreement with the simulation CT. The green ROI in **Figure 3** was used for SSIM analysis, and the red ROI for RMSE calculation.

**Figure 4:**
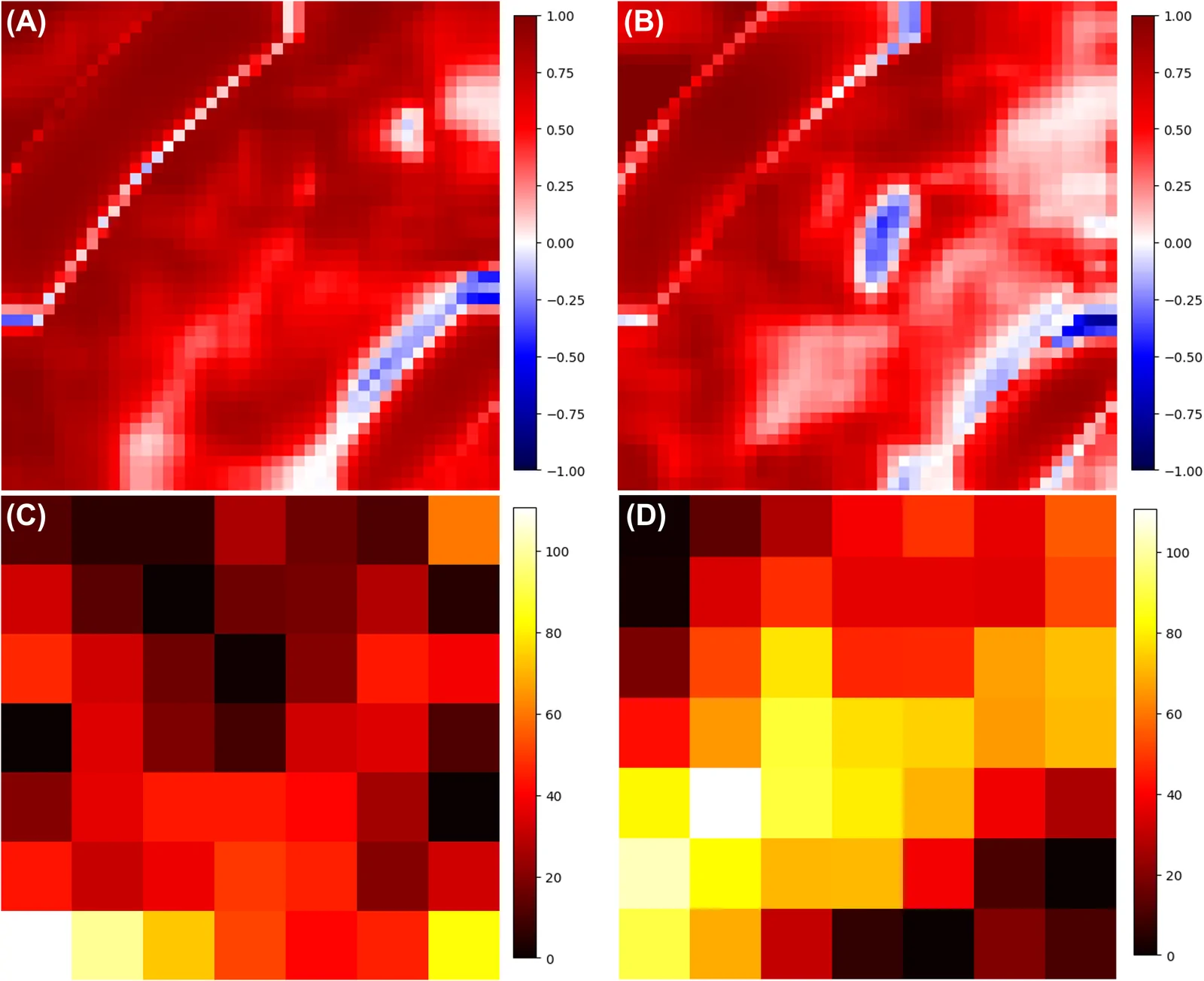
SSIM maps and RMSE difference maps of HyperSight and Halcyon 3.1 using Simulation CT as the reference. **(A, B)** SSIM maps, and **(C, D)** RMSE difference maps.

**Figure 4** further compares the modalities using SSIM maps and RMSE difference maps. HyperSight showed generally higher SSIM values across most regions—appearing predominantly as warmer colors (red/yellow)—with only modest variations near the lung–soft tissue interfaces, indicating better structural preservation relative to CT. In comparison, Halcyon 3.1 demonstrated lower SSIM values over broader soft-tissue areas, represented by cooler colors (blue), although the degree of reduction varied spatially rather than forming uniform regions of loss. RMSE difference maps revealed the same trend: HyperSight showed low-magnitude, spatially uniform error, whereas Halcyon 3.1 displayed irregularly distributed high-error regions.

Quantitatively, HyperSight significantly outperformed Halcyon 3.1 across all structural similarity metrics (Wilcoxon signed-rank test, p < 0.05). SSIM was higher for HyperSight (0.664) than Halcyon 3.1 (0.513), with a paired mean difference of 0.152 (95% CI, 0.087–0.216) and a rank-biserial correlation of 1.00. Both RMSE (35.9 vs. 52.4) and NRMSE (0.418 vs. 0.605) were significantly lower. Among the SSIM components, the Structure term showed a significant advantage for HyperSight (0.935 vs. 0.899, p < 0.05), whereas Luminance and Contrast exhibited similar trends but did not reach statistical significance (p > 0.05). Detailed pairwise comparison statistics, including paired mean differences, 95% confidence intervals, effect sizes, and p-values, for all quantitative metrics are provided in **Supplementary Table S1**.

### 3.2 Uniformity metrics

**Figure 5:**
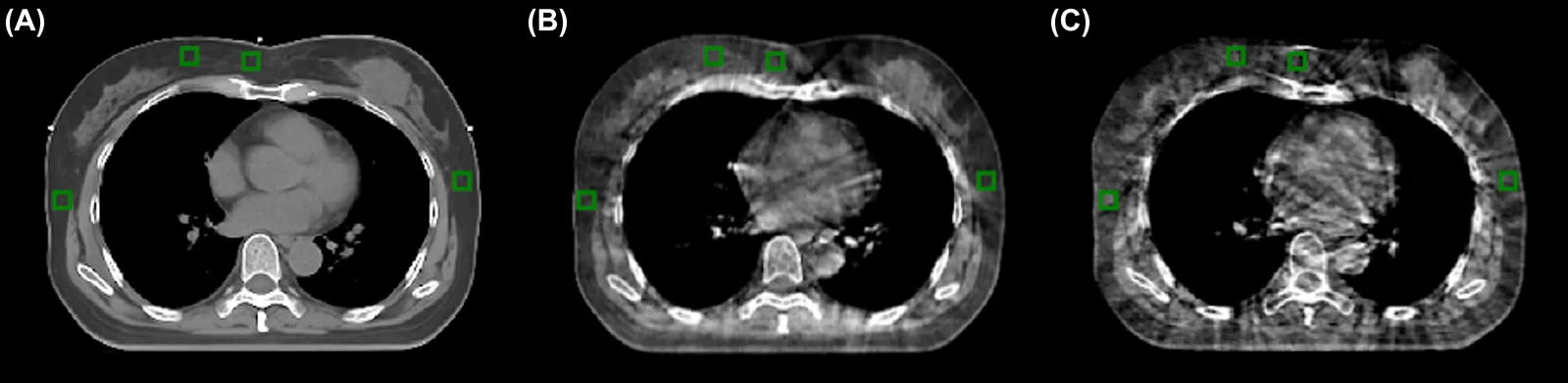
Uniformity measurement with 4 ROIs (green box, 10 × 10 mm): **(A)** simulation CT, **(B)** HyperSight, and **(C)** Halcyon 3.1.

Uniformity was quantified using four 10×10 mm ROIs (**Figure 5**). Mean HU uniformity did not differ significantly between simulation CT (-89.02) and HyperSight (-91.24) (p = 0.473). In contrast, Halcyon 3.1 showed significantly poorer uniformity relative to both CT (-117.33, p = 0.006) and HyperSight (p = 0.010).

Uniformity standard deviation further highlighted the differences: simulation CT (7.02) demonstrated the most stable uniformity, while HyperSight (20.74) showed moderate variability and Halcyon 3.1 (32.58) exhibited the greatest non-uniformity. All pairwise comparisons were statistically significant (p < 0.01).

### 3.3 Signal and contrast metrics (signal, SNR, contrast, CNR)

**Figure 6:**
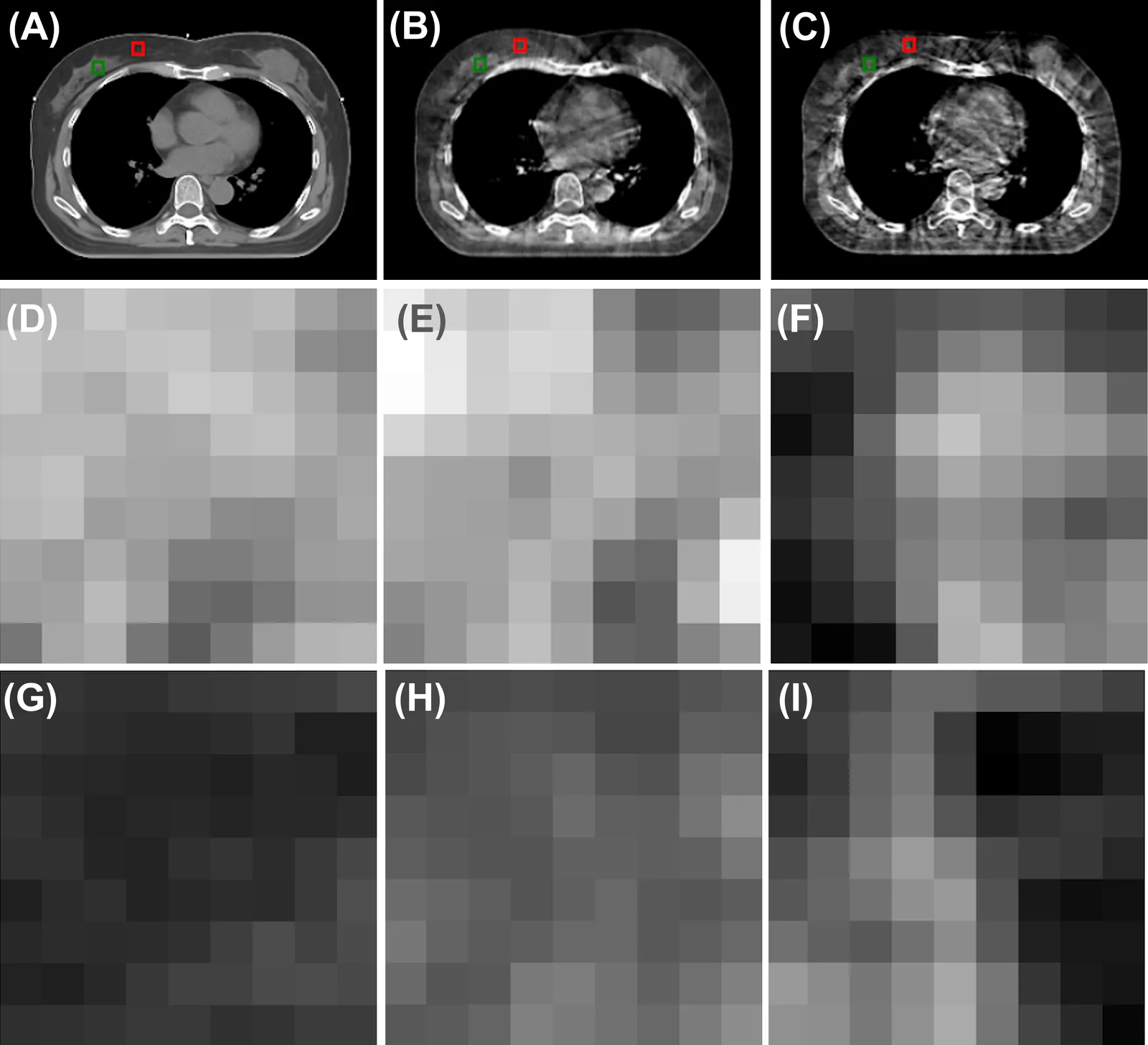
Comparison of ROI images, signal maps, and noise maps for Simulation CT, HyperSight, and Halcyon 3.1. **(A–C)** ROI locations [green box (10 × 10 mm) for signal and red box (10 × 10 mm) for noise], **(D–F)** signal maps, and **(G–I)** noise maps.

As illustrated in **Figure 6**, simulation CT exhibited stable and spatially consistent signal and noise characteristics, whereas Halcyon 3.1 demonstrated large fluctuations in both metrics. HyperSight produced intermediate but substantially improved values compared with Halcyon 3.1.

Numerically, simulation CT showed significantly higher signal intensity compared with Halcyon 3.1 (3.79 vs. -22.30, t-test p = 0.032). No significant difference was found between simulation CT and HyperSight (p = 0.222).

SNR values did not differ significantly across modalities (p > 0.05).

Contrast was slightly higher for Halcyon 3.1 (115.55) compared with HyperSight (100.02) and simulation CT (104.25), though none of these differences reached statistical significance (p > 0.05).

CNR, however, showed clear divergence. Simulation CT (9.91) had significantly higher CNR than Halcyon 3.1 (3.61, *p* = 0.002), with a paired mean difference of 6.30 (95% CI, 2.95–9.65; Cohen’s dz = 1.20). HyperSight (7.94) also showed significantly higher CNR than Halcyon 3.1 (*p* = 0.001), with a paired mean difference of 4.33 (95% CI, 2.13–6.53; Cohen’s dz = 1.25). No significant difference was observed between simulation CT and HyperSight (*p* = 0.133), indicating comparable contrast-to-noise performance between HyperSight and simulation CT.

### 3.4 Edge-based metrics (EPI, EPS)

**Figure 7:**
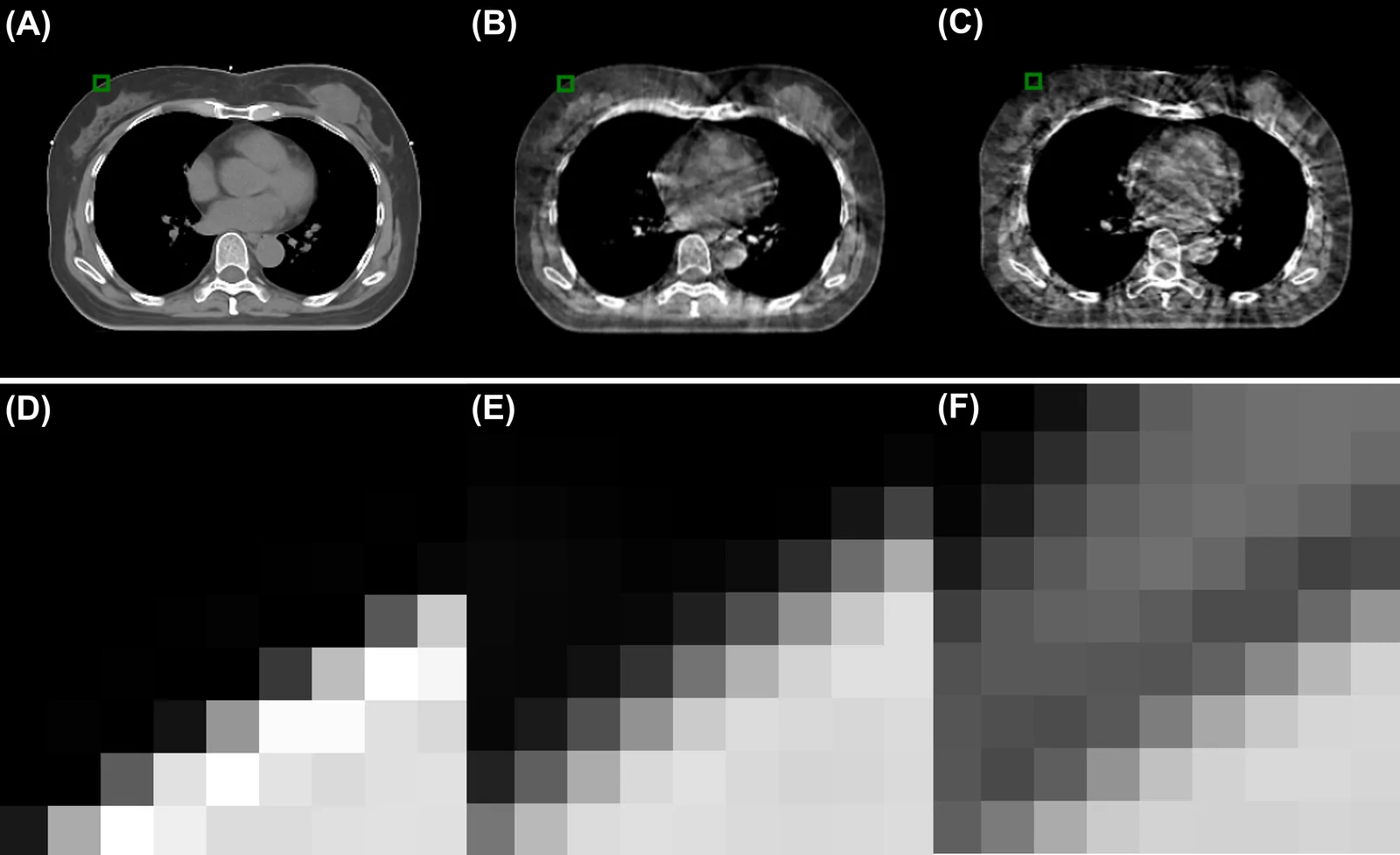
Edge images of Simulation CT, HyperSight, and Halcyon 3.1. **(A–C)** Edge ROI locations [green box (10 × 10 mm)], and **(D–F)** magnified ROI images of the edge region.

**Figure 7** shows edge ROIs used for evaluating boundary characteristics. EPI values did not differ significantly among the three modalities (p > 0.05).

In contrast, EPS revealed clear differences. Simulation CT (447.53) had significantly higher EPS values than HyperSight (400.21, p = 0.002), with a paired mean difference of 47.32 (95% CI, 21.45–73.19; Cohen’s dz = 1.16), and Halcyon 3.1 (310.19, p < 0.001). HyperSight also showed significantly higher EPS values than Halcyon 3.1 (p < 0.001), with a paired mean difference of 90.02 (95% CI, 54.93–125.12; Cohen’s dz = 1.63). These findings indicate that simulation CT and HyperSight exhibited reduced edge blurring and better preservation of edge-intensity transitions than Halcyon 3.1, reflecting superior stability in boundary signal reproduction.

### 3.5 Electron density curve analysis

**Figure 8:**
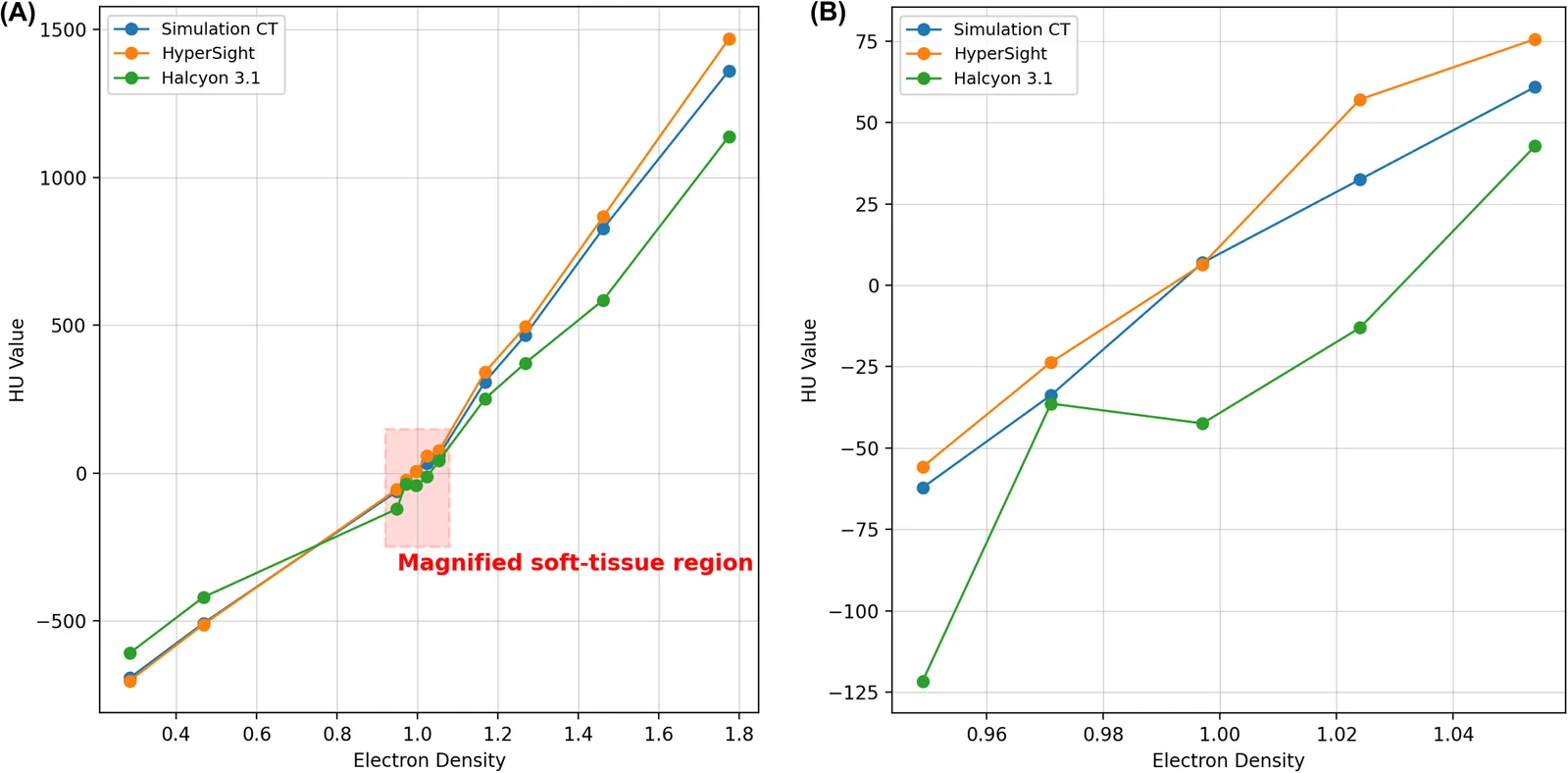
Electron density (ED) curves for each modality: **(A)** full range and **(B)** magnified soft-tissue range.

**Figure 8A** shows the HU-to-ED relationships for all modalities. Simulation CT and HyperSight demonstrated nearly identical linearity across ρ_e_ = 0.285–1.774. Halcyon 3.1, however, exhibited substantial deviations: elevated HU values in lung-equivalent inserts, suppressed HU values in soft tissues, and large divergence in high-density bone-equivalent regions (ρ_e_ > 1.168).

The expanded soft-tissue view (**Figure 8B**) further emphasized this pattern. Solid water inserts (ρ_e_ ≈ 0.997) yielded HU values of 6.9 (SD 3.1) for simulation CT and 6.4 (SD 4.1) for HyperSight, both well within the expected range (0 ± 10 HU). Halcyon 3.1, however, produced HU = -42.5 with SD = 47.5, indicating both severe HU bias and large variability.

### 3.6 Dosimetric evaluation

Comparison of the three plan types revealed that simulation CT produced the highest conformity and homogeneity. CI_Paddick was greatest for simulation CT (0.903 ± 0.034), followed by HyperSight (0.883 ± 0.053), and lowest for Halcyon 3.1 (0.840 ± 0.066). All pairwise comparisons were statistically significant (Halcyon 3.1 vs. HyperSight p = 0.003; Halcyon 3.1 vs. CT p = 0.001; HyperSight vs. CT p = 0.003). The difference between simulation CT and Halcyon 3.1 was associated with a paired mean difference of 0.063 (95% CI, 0.032–0.095) and a large effect size (Cohen’s dz = 1.28).

HI showed a consistent pattern: simulation CT produced the most homogeneous dose distribution (0.108 ± 0.011), HyperSight maintained similar performance (0.126 ± 0.026), and Halcyon 3.1 showed the least homogeneous distribution (0.166 ± 0.062). All modality comparisons were statistically significant (p < 0.01).

**Figure 9:**
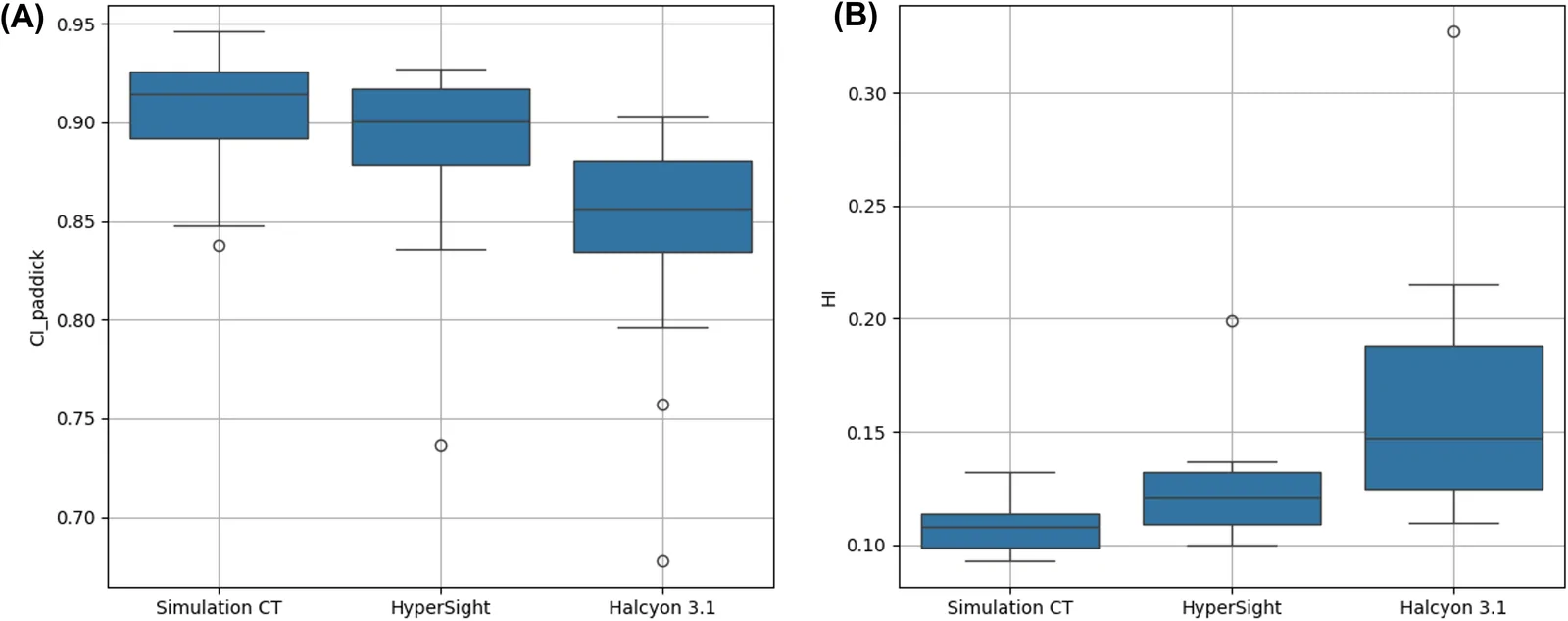
Box plots for each modality: **(A)** CI and **(B)** HI.

Box-plot visualization (**Figure 9**) confirmed these results: CT and HyperSight formed tight, high-quality clusters for both CI and HI, whereas Halcyon 3.1 showed lower medians and much wider dispersion. These results indicate that HyperSight-based CBCT dose recalculation yielded plan quality substantially closer to simulation CT than to Halcyon 3.1.

Additional DVH-based analysis demonstrated that HyperSight showed smaller deviations in PTV coverage relative to the simulation CT than Halcyon. HyperSight showed reductions in D95 (-21 cGy; Cohen’s dz = -0.68, p = 0.001), D98 (-51 cGy; Cohen’s dz = -0.76, p < 0.001), and V95%Rx (-0.5%; Cohen’s dz = -0.67, p < 0.001). Corresponding reductions for Halcyon were larger, with D95 reduced by 157 cGy (Cohen’s dz = -1.01, p = 0.001), D98 by 353 cGy (Cohen’s dz = -0.93, p < 0.001), and V95%Rx by 3.0% (Cohen’s dz = -1.06, p < 0.001). For the ipsilateral lung, heart, contralateral breast, and maximum dose, no statistically significant differences were observed between either CBCT modality and the simulation CT.

### 3.7 Adaptive target volume changes

**Figure 10:**
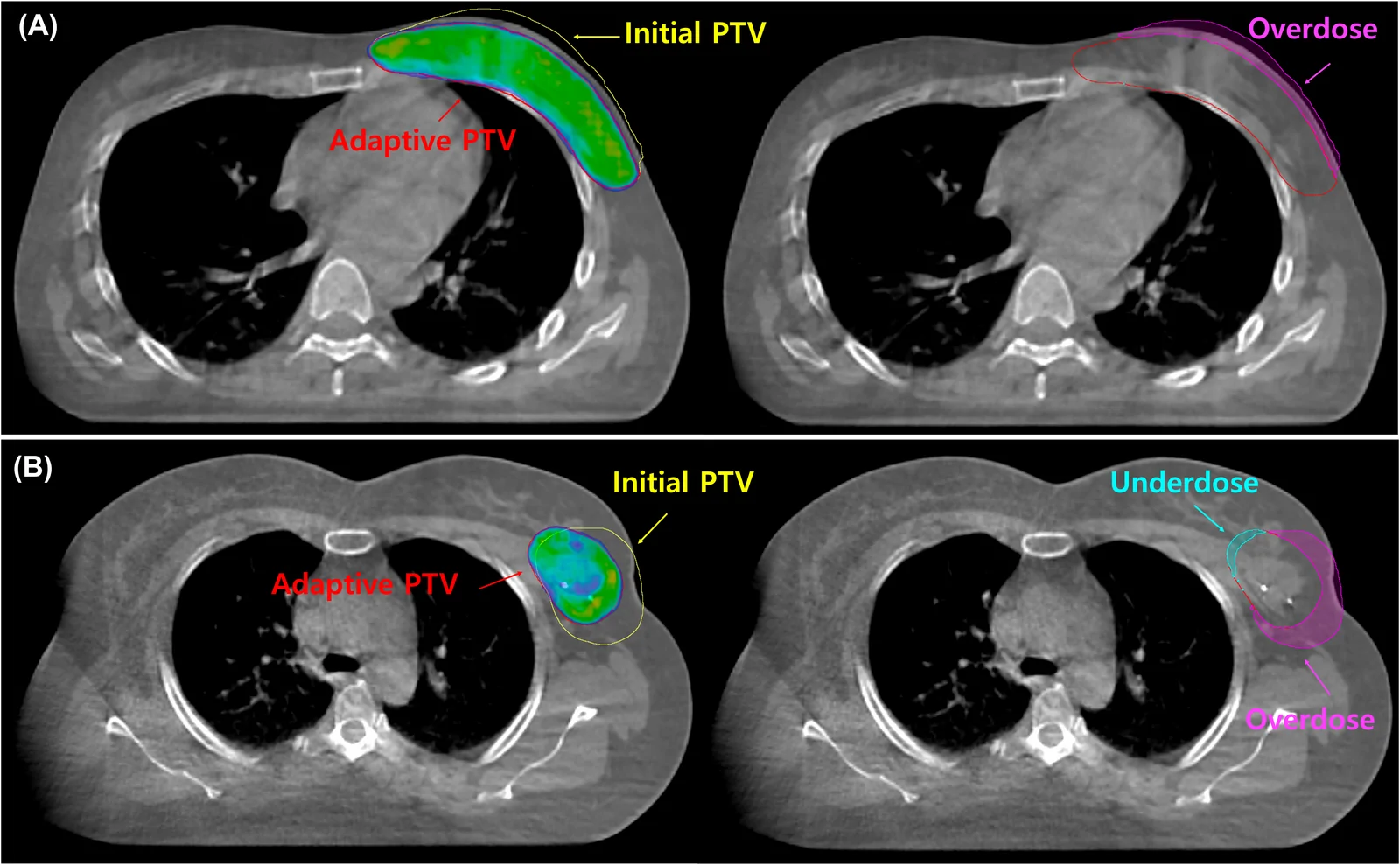
Differences in PTV between the initial and adaptive plans, showing overdose and underdose volumes: **(A)** whole breast and **(B)** tumor bed boost region.

**Figure 10** illustrates representative examples of target-volume changes observed during treatment and the resulting spatial mismatch between the initial and adaptive target volumes. If the original treatment plan had been maintained without adaptation, regions included in the initial PTV but excluded from the adaptive PTV would have continued to receive unnecessary prescription dose (hot regions), whereas regions newly included in the adaptive PTV would have been insufficiently covered (cold regions).

Across the 12 patients, hot regions (initial PTV - adaptive PTV) were identified in 11 of 12 patients, with a mean volume of 41.2 cm³ (range, 0.02–114.6 cm³). Cold regions (adaptive PTV - initial PTV) were observed in all 12 patients, with a mean volume of 24.1 cm³ (range, 0.01–117.1 cm³).

## 4 Discussion

This study evaluated the HU accuracy, image quality, and dosimetric applicability of the newly integrated HyperSight CBCT on the Halcyon platform and compared its performance with Halcyon 3.1 and simulation CT to determine its feasibility for offline ART in breast cancer. Following HyperSight implementation at our institution, frequent anatomical and volumetric changes, including seroma shrinkage and tumor bed migration, were consistently observed during treatment. Such changes directly impact treatment accuracy by changing the target volume defined at simulation, potentially increasing unnecessary dose to normal tissues or compromising target coverage when the original plan is maintained throughout the course of radiotherapy.

HyperSight-based offline adaptive assessment demonstrated that clinically meaningful anatomical deviations frequently develop during breast radiotherapy. Quantitative comparison between the initial and adaptive target volumes revealed substantial spatial mismatch in most patients, indicating that maintaining the original treatment plan throughout the treatment course may result in unnecessary irradiation of normal tissue as well as insufficient coverage of the updated target volume. These anatomical changes were most likely attributable to seroma shrinkage, soft-tissue deformation, and daily setup variability. Compared with conventional CBCT, the improved soft-tissue contrast, HU stability, and edge definition of HyperSight may allow these anatomical changes to be visualized and quantified with greater confidence. Taken together, these findings suggest that HyperSight may be useful for identifying clinically relevant dosimetric changes and may provide a practical option for periodic offline adaptive radiotherapy to help maintain target coverage while minimizing unnecessary irradiation of normal tissues.

The baseline image-quality performance of HyperSight has been reported in prior phantom-based evaluations (12, 26, 27), and was confirmed at our institution during commissioning using standard phantoms (ACR 464 and Catphan 604). Building on this system-level validation, the present study focused on the clinical applicability of HyperSight in actual breast-cancer patients. Because the image-quality analysis in this study was performed using patient CBCT rather than static phantom images, clinical variability required careful methodological control. To reduce confounding factors, we evaluated structurally stable parenchymal regions in the contralateral breast and applied a standardized workflow—global rigid registration, local refinement, and line-profile–based micro-alignment—to minimize anatomical discrepancies across modalities. ROIs were placed in identical anatomical locations for all imaging modalities, and areas minimally affected by respiratory motion were selected to ensure fair comparison.

All CBCT scans were acquired under free-breathing conditions. Consequently, motion-related artifacts were unavoidable, particularly in Halcyon 3.1, which has a longer acquisition time and is therefore more susceptible to respiratory blurring and streak artifacts. Respiratory motion has been shown to degrade target delineation and dose-calculation accuracy even with rapid-acquisition HyperSight CBCT (28). HyperSight, by contrast, acquires images within approximately 6 seconds, making breath-hold acquisitions clinically feasible. Breath-hold HyperSight imaging is expected to further reduce motion artifacts and yield CBCT quality approaching that of simulation CT (29). Although only free-breathing data were analyzed in this study, breath-hold HyperSight protocols may provide a meaningful improvement in quantitative image accuracy in routine practice.

HyperSight also demonstrated HU-to-ED linearity that closely matched simulation CT, while Halcyon 3.1 showed noticeable deviations in this relationship, particularly within the soft-tissue density range. These deviations in Halcyon 3.1 reduced the accuracy of HU-to-ED conversion. In contrast, HyperSight’s calibration approach, which incorporates multi-kVp spectrum correction and QUART-based water-equivalent referencing, effectively minimized energy-dependent HU bias and reduced systemic calibration error. Our soft-tissue HU results are consistent with previous phantom-based reports: HyperSight yielded solid-water HU values within the accepted water tolerance (6.4 HU, SD 4.1), comparable to the close CT agreement reported by Nelson et al., who observed HU differences within approximately 30 HU for near-water-equivalent materials (16), and by Sijtsema et al., who reported agreement within 35 HU across calibration-phantom inserts (30). This concordance across independent studies supports the reliability of the HU-to-ED conversion used for our CBCT-based dose recalculation.

Notably, HyperSight achieved improved image quality despite a substantially lower CTDI_vol than Halcyon 3.1 (3.39 vs. 6.04 mGy). This apparent paradox reflects the dose efficiency of the HyperSight platform rather than a dependence on imaging dose. Rather than relying on increased photon fluence, HyperSight improves image quality through a high-efficiency detector with a novel CsI scintillator and low-noise electronics, a high-ratio anti-scatter grid combined with hardware-based scatter correction, and a statistical iterative reconstruction algorithm incorporating Acuros-based scatter modeling (12, 29). These features jointly suppress scatter and noise while preserving soft-tissue contrast, allowing high image quality to be maintained or even improved at a reduced imaging dose. We also acknowledge that differences in acquisition protocols between the two systems, including tube-current (mAs) settings, may have contributed to the observed results; however, these protocol settings represent the clinically implemented, vendor-recommended configurations for each platform, and the improvements are consistent with the intrinsic dose-efficiency advantages reported for HyperSight in previous studies (31).

In our study, CBCT-based dose recalculation using HyperSight reproduced CT-based CI_Paddick and HI with high accuracy, indicating improved dosimetric consistency compared with conventional CBCT. Additional DVH analysis further demonstrated that the principal dosimetric differences were confined to PTV coverage. Although statistically significant differences were observed for D95, D98, and V95%Rx, the absolute deviations remained small, whereas no statistically significant differences were observed for the ipsilateral lung, heart, contralateral breast, or maximum dose.

This dosimetric agreement is in line with prior HyperSight studies: Nelson et al. reported dose-calculation accuracy within 1% of simulation CT with 3D gamma pass rates ≥90% (1%/1 mm) on anthropomorphic phantoms (16), and a recent multi-institutional validation by Lin et al. demonstrated sub-2% dosimetric deviations and strong agreement with CT across three centers (32). Our reproduction of CT-based CI_Paddick and HI in breast patients is consistent with these phantom- and multi-institution-based findings, extending them to a clinical breast-cancer cohort. These findings suggest that HyperSight-based offline ART may serve as one viable option for CBCT-guided adaptive assessment in breast radiotherapy, where progressive changes in breast and tumor-bed volume are common (33). Because HyperSight can quantitatively detect these anatomical deviations, it may serve as a practical tool for identifying clinically relevant overdosed or underdosed regions and for guiding timely adaptive plan updates. Such adaptive correction can reduce unnecessary irradiation of normal tissues while preserving adequate target coverage.

Although HyperSight may offer offline adaptation as a potential alternative to repeat simulation CT in selected cases, it still requires clinicians and radiation therapists to routinely evaluate CBCT images and determine when adaptation is warranted. This underscores the need for institutional standardization of imaging surveillance and adaptive triggers. As this is an early, preliminary study conducted in limited cases, several directions remain for future work. Prospective, multi-fraction evaluations that track anatomical and dosimetric changes across the treatment course would help clarify when adaptation is clinically warranted. In addition, direct validation of CBCT-based dose calculation for example, against repeat simulation CT, synthetic CT, or independent dose measurements together with studies across additional anatomical sites such as head and neck or pelvis, will be needed to further establish the robustness and generalizability of HU-to-ED–based adaptive workflows using HyperSight (34, 35).

This study has several limitations. First, this was a retrospective feasibility analysis based on a limited cohort of 12 patients, which precludes definitive conclusions regarding routine clinical implementation. Second, because Halcyon 3.1 and HyperSight images were acquired on different days, residual differences in respiration, positioning, and breast anatomy could not be fully eliminated. To minimize this, only image sets acquired within ±1 day were included, and structurally stable parenchymal regions were selected for evaluation; nevertheless, despite the standardized registration and ROI-control workflow, these factors may have affected SSIM, RMSE, and other image-quality parameters. Third, all analyses were performed under free-breathing conditions, and CBCT-based dose recalculation was not independently verified. Finally, because this study was exploratory and included a relatively small sample size, formal multiple-comparison correction was not applied. Accordingly, the statistical findings should be interpreted in conjunction with the reported effect sizes and 95% confidence intervals. These limitations should be considered when interpreting the present findings, which are best regarded as hypothesis-generating rather than confirmatory.

## 5 Conclusion

Our clinical analysis demonstrated that anatomical changes and volumetric variations occur frequently during breast radiotherapy, indicating that maintaining the initial treatment plan throughout the entire course may compromise dosimetric accuracy. These findings support the potential value of periodic assessment for offline ART in breast cancer treatment.

We evaluated whether HyperSight provides the image accuracy and dosimetric accuracy required to support such an offline ART workflow. Compared with Halcyon 3.1, HyperSight showed clear overall improvements in image quality and HU consistency, achieving a level of accuracy that closely aligned with simulation CT. These improvements supported more reliable CBCT-based dose recalculation, with dosimetric performance remaining largely consistent with the simulation CT.

In conclusion, our results suggest that HyperSight provides image quality and HU-to-ED reliability closer to simulation CT than Halcyon 3.1, which may support CBCT-based dose reassessment and offline plan adaptation. HyperSight may also help reduce reliance on repeat simulation CT in selected cases. However, as this was a preliminary feasibility study involving a limited cohort of 12 patients, further validation against repeat simulation CT, synthetic CT, or independent dose measurements is needed before routine clinical implementation.

## Data Availability

The raw data supporting the conclusions of this article will be made available by the authors, without undue reservation.
