# Supplementary material for: A clinical evaluation of HyperSight CBCT enhanced offline adaptive breast radiotherapy
Source: Front Oncol. 2026 Aug 19;16:1912819. doi: 10.3389/fonc.2026.1912819 (PMC13533814; doi:10.3389/fonc.2026.1912819)
Supplement: Supplementary file 1 [file Table1.docx]

| **Supplementary Table S1. Pairwise comparison statistics for quantitative imaging and dosimetric metrics.** | | | | | |
| --- | --- | --- | --- | --- | --- |
|  |  |  |  |  |  |
| **Metric** | **Comparison** | **Mean difference** | **95% CI** | **Effect size** | **P-value** |
| ***Structural similarity metrics*** | | | | | |
| SSIM | HyperSight vs Halcyon 3.1 | 0.152 | 0.087 to 0.216 | Rank-biserial correlation = 1.000 | <0.001 |
| RMSE | HyperSight vs Halcyon 3.1 | -16.473 | -30.543 to -2.403 | Rank-biserial correlation = -0.821 | 0.009 |
| NRMSE | HyperSight vs Halcyon 3.1 | -0.187 | -0.357 to -0.017 | Rank-biserial correlation = -0.821 | 0.009 |
| SSIM Luminance | HyperSight vs Halcyon 3.1 | 0.012 | -0.003 to 0.027 | Rank-biserial correlation = 0.487 | 0.151 |
| SSIM Contrast | HyperSight vs Halcyon 3.1 | 0.001 | -0.001 to 0.003 | Cohen's dz = 0.320 | 0.291 |
| SSIM Structure | HyperSight vs Halcyon 3.1 | 0.036 | 0.004 to 0.068 | Rank-biserial correlation = 0.718 | 0.027 |
| ***Uniformity metrics*** | | | | | |
| ROI1 Mean | HyperSight vs Halcyon 3.1 | 25.961 | -7.219 to 59.141 | Cohen's dz = 0.497 | 0.113 |
| ROI1 Mean | Simulation CT vs Halcyon 3.1 | 25.475 | -7.215 to 58.165 | Cohen's dz = 0.495 | 0.114 |
| ROI1 Mean | Simulation CT vs HyperSight | -0.486 | -16.734 to 15.763 | Cohen's dz = -0.019 | 0.949 |
| ROI2 Mean | HyperSight vs Halcyon 3.1 | 36.847 | 17.893 to 55.800 | Cohen's dz = 1.235 | 0.001 |
| ROI2 Mean | Simulation CT vs Halcyon 3.1 | 32.126 | 7.351 to 56.900 | Cohen's dz = 0.824 | 0.016 |
| ROI2 Mean | Simulation CT vs HyperSight | -4.721 | -21.839 to 12.398 | Cohen's dz = -0.175 | 0.556 |
| ROI3 Mean | HyperSight vs Halcyon 3.1 | 26.963 | 0.881 to 53.044 | Cohen's dz = 0.657 | 0.044 |
| ROI3 Mean | Simulation CT vs Halcyon 3.1 | 27.046 | 6.285 to 47.806 | Cohen's dz = 0.828 | 0.015 |
| ROI3 Mean | Simulation CT vs HyperSight | 0.083 | -19.387 to 19.554 | Cohen's dz = 0.003 | 0.993 |
| ROI4 Mean | HyperSight vs Halcyon 3.1 | 14.584 | -27.531 to 56.700 | Cohen's dz = 0.220 | 0.462 |
| ROI4 Mean | Simulation CT vs Halcyon 3.1 | 28.612 | -9.960 to 67.184 | Cohen's dz = 0.471 | 0.131 |
| ROI4 Mean | Simulation CT vs HyperSight | 14.028 | 0.353 to 27.702 | Cohen's dz = 0.652 | 0.045 |
| Overall Mean | HyperSight vs Halcyon 3.1 | 26.089 | 7.506 to 44.673 | Cohen's dz = 0.892 | 0.010 |
| Overall Mean | Simulation CT vs Halcyon 3.1 | 28.313 | 9.769 to 46.858 | Cohen's dz = 0.970 | 0.006 |
| Overall Mean | Simulation CT vs HyperSight | 2.224 | -4.358 to 8.806 | Cohen's dz = 0.215 | 0.473 |
| Overall STD | HyperSight vs Halcyon 3.1 | -11.841 | -19.989 to -3.693 | Cohen's dz = -0.923 | 0.008 |
| Overall STD | Simulation CT vs Halcyon 3.1 | -25.563 | -34.044 to -17.083 | Cohen's dz = -1.915 | <0.001 |
| Overall STD | Simulation CT vs HyperSight | -13.722 | -21.064 to -6.381 | Cohen's dz = -1.188 | 0.002 |
| ***Signal and contrast metrics*** | | | | | |
| Signal Mean | HyperSight vs Halcyon 3.1 | 18.944 | -2.597 to 40.486 | Cohen's dz = 0.559 | 0.079 |
| Signal Mean | Simulation CT vs Halcyon 3.1 | 26.086 | 2.633 to 49.540 | Cohen's dz = 0.707 | 0.032 |
| Signal Mean | Simulation CT vs HyperSight | 7.142 | -4.989 to 19.274 | Cohen's dz = 0.374 | 0.222 |
| Noise Mean | HyperSight vs Halcyon 3.1 | 30.969 | -2.329 to 64.268 | Cohen's dz = 0.591 | 0.065 |
| Noise Mean | Simulation CT vs Halcyon 3.1 | 33.887 | 4.642 to 63.133 | Cohen's dz = 0.736 | 0.027 |
| Noise Mean | Simulation CT vs HyperSight | 2.918 | -15.007 to 20.843 | Rank-biserial correlation = 0.154 | 0.677 |
| SNR | HyperSight vs Halcyon 3.1 | 0.155 | -1.038 to 1.347 | Cohen's dz = 0.082 | 0.781 |
| SNR | Simulation CT vs Halcyon 3.1 | 0.714 | -0.621 to 2.049 | Cohen's dz = 0.340 | 0.264 |
| SNR | Simulation CT vs HyperSight | 0.559 | -0.850 to 1.969 | Cohen's dz = 0.252 | 0.401 |
| Contrast | HyperSight vs Halcyon 3.1 | -15.521 | -58.792 to 27.750 | Cohen's dz = -0.228 | 0.447 |
| Contrast | Simulation CT vs Halcyon 3.1 | -11.297 | -44.222 to 21.628 | Cohen's dz = -0.218 | 0.466 |
| Contrast | Simulation CT vs HyperSight | 4.224 | -15.692 to 24.140 | Cohen's dz = 0.135 | 0.650 |
| CNR | HyperSight vs Halcyon 3.1 | 4.332 | 2.135 to 6.529 | Cohen's dz = 1.253 | 0.001 |
| CNR | Simulation CT vs Halcyon 3.1 | 6.301 | 2.952 to 9.649 | Cohen's dz = 1.196 | 0.002 |
| CNR | Simulation CT vs HyperSight | 1.969 | -0.699 to 4.636 | Cohen's dz = 0.469 | 0.133 |
| ***Edge-based metrics*** | | | | | |
| EPI | HyperSight vs Halcyon 3.1 | -8.262 | -98.887 to 82.363 | Cohen's dz = -0.058 | 0.845 |
| EPI | Simulation CT vs Halcyon 3.1 | -5.865 | -102.352 to 90.621 | Cohen's dz = -0.039 | 0.896 |
| EPI | Simulation CT vs HyperSight | 2.397 | -57.772 to 62.565 | Rank-biserial correlation = 0.154 | 0.677 |
| EPS | HyperSight vs Halcyon 3.1 | 90.025 | 54.932 to 125.117 | Cohen's dz = 1.630 | <0.001 |
| EPS | Simulation CT vs Halcyon 3.1 | 137.343 | 98.116 to 176.569 | Cohen's dz = 2.225 | <0.001 |
| EPS | Simulation CT vs HyperSight | 47.318 | 21.448 to 73.187 | Cohen's dz = 1.162 | 0.002 |
| ***Dosimetric metrics*** | | | | | |
| CI_Paddick | HyperSight vs Halcyon 3.1 | 0.044 | 0.018 to 0.069 | Cohen's dz = 1.09 | 0.003 |
| CI_Paddick | Simulation CT vs Halcyon 3.1 | 0.063 | 0.032 to 0.095 | Cohen's dz = 1.28 | 0.001 |
| CI_Paddick | Simulation CT vs HyperSight | 0.020 | 0.002 to 0.037 | Rank-biserial correlation = 0.69 | 0.003 |
| HI | HyperSight vs Halcyon 3.1 | -0.040 | -0.080 to -0.000 | Cohen's dz = -0.64 | <0.01 |
| HI | Simulation CT vs Halcyon 3.1 | -0.058 | -0.097 to -0.019 | Cohen's dz = -0.95 | <0.01 |
| HI | Simulation CT vs HyperSight | -0.018 | -0.033 to -0.003 | Rank-biserial correlation = -0.56 | <0.01 |
| ***DVH-based dosimetric metrics*** | | | | | |
| PTV Dmean | HyperSight vs Simulation CT | 7.80 | -8.0 to 23.7 | Cohen's dz = 0.31 | 0.622 |
| PTV Dmean | Halcyon 3.1 vs Simulation CT | -33.40 | -57.6 to -9.2 | Cohen's dz = -0.88 | 0.021 |
| PTV D95 | HyperSight vs Simulation CT | -21.30 | -41.1 to -1.4 | Cohen's dz = -0.68 | 0.001 |
| PTV D95 | Halcyon 3.1 vs Simulation CT | -156.70 | -255.0 to -58.4 | Cohen's dz = -1.01 | 0.001 |
| PTV D98 | HyperSight vs Simulation CT | -51.00 | -93.9 to -8.2 | Cohen's dz = -0.76 | <0.001 |
| PTV D98 | Halcyon 3.1 vs Simulation CT | -353.00 | -595.4 to -110.7 | Cohen's dz = -0.93 | <0.001 |
| PTV D2 | HyperSight vs Simulation CT | 23.90 | -1.3 to 49.1 | Cohen's dz = 0.60 | 0.064 |
| PTV D2 | Halcyon 3.1 vs Simulation CT | 19.10 | 3.0 to 35.2 | Cohen's dz = 0.75 | 0.021 |
| PTV D50 | HyperSight vs Simulation CT | 13.40 | -4.1 to 31.0 | Cohen's dz = 0.49 | 0.151 |
| PTV D50 | Halcyon 3.1 vs Simulation CT | -4.10 | -12.5 to 4.3 | Cohen's dz = -0.31 | 0.380 |
| PTV V95%Rx | HyperSight vs Simulation CT | -0.52 | -1.0 to -0.0 | Cohen's dz = -0.67 | <0.001 |
| PTV V95%Rx | Halcyon 3.1 vs Simulation CT | -2.98 | -4.8 to -1.2 | Cohen's dz = -1.06 | <0.001 |
| PTV V107%Rx | HyperSight vs Simulation CT | 2.05 | -1.8 to 5.9 | Cohen's dz = 0.34 | 0.339 |
| PTV V107%Rx | Halcyon 3.1 vs Simulation CT | -1.50 | -4.5 to 1.5 | Cohen's dz = -0.31 | 0.569 |
| Ipsilateral lung Dmean | HyperSight vs Simulation CT | 22.30 | -8.8 to 53.3 | Cohen's dz = 0.46 | 0.176 |
| Ipsilateral lung Dmean | Halcyon 3.1 vs Simulation CT | -0.10 | -30.9 to 30.7 | Cohen's dz = -0.00 | 0.470 |
| Ipsilateral lung V5Gy | HyperSight vs Simulation CT | 1.03 | 0.2 to 1.9 | Cohen's dz = 0.76 | 0.052 |
| Ipsilateral lung V5Gy | Halcyon 3.1 vs Simulation CT | 0.21 | -2.1 to 2.5 | Cohen's dz = 0.06 | 0.519 |
| Ipsilateral lung V10Gy | HyperSight vs Simulation CT | 0.95 | -0.1 to 2.0 | Cohen's dz = 0.56 | 0.052 |
| Ipsilateral lung V10Gy | Halcyon 3.1 vs Simulation CT | -0.01 | -1.2 to 1.1 | Cohen's dz = -0.01 | 0.519 |
| Ipsilateral lung V20Gy | HyperSight vs Simulation CT | 0.52 | -0.6 to 1.7 | Cohen's dz = 0.29 | 0.266 |
| Ipsilateral lung V20Gy | Halcyon 3.1 vs Simulation CT | -0.10 | -0.6 to 0.4 | Cohen's dz = -0.14 | 0.733 |
| Heart Dmean | HyperSight vs Simulation CT | 1.30 | -3.0 to 5.8 | Cohen's dz = 0.24 | 0.652 |
| Heart Dmean | Halcyon 3.1 vs Simulation CT | -0.40 | -5.9 to 5.1 | Cohen's dz = -0.05 | 0.652 |
| Heart Dmax | HyperSight vs Simulation CT | 3.30 | -33.3 to 39.8 | Cohen's dz = 0.07 | 0.359 |
| Heart Dmax | Halcyon 3.1 vs Simulation CT | -12.10 | -34.4 to 10.2 | Cohen's dz = -0.42 | 0.359 |
| Contralateral breast Dmean | HyperSight vs Simulation CT | -0.20 | -7.7 to 7.4 | Cohen's dz = -0.01 | 0.232 |
| Contralateral breast Dmean | Halcyon 3.1 vs Simulation CT | 2.60 | -21.9 to 27.1 | Cohen's dz = 0.08 | 0.695 |
| Contralateral breast Dmax | HyperSight vs Simulation CT | 2.60 | -13.2 to 18.5 | Cohen's dz = 0.12 | 0.846 |
| Contralateral breast Dmax | Halcyon 3.1 vs Simulation CT | 1.40 | -12.9 to 15.6 | Cohen's dz = 0.07 | 1.000 |
| Contralateral breast V5Gy | HyperSight vs Simulation CT | 0.23 | -0.3 to 0.7 | Cohen's dz = 0.34 | 0.375 |
| Contralateral breast V5Gy | Halcyon 3.1 vs Simulation CT | 0.60 | -1.2 to 2.4 | Cohen's dz = 0.24 | 0.846 |
| Global Dmax | HyperSight vs Simulation CT | -5.80 | -43.8 to 32.0 | Cohen's dz = -0.10 | 0.970 |
| Global Dmax | Halcyon 3.1 vs Simulation CT | 21.50 | -20.2 to 63.1 | Cohen's dz = 0.33 | 0.339 |
| *Abbreviations: CI, confidence interval; CI_Paddick, Paddick conformity index; HI, homogeneity index; PTV, planning target volume; Dmean, mean dose; Dmax, maximum dose; Dx, dose received by x% of the structure volume; VxGy, percentage of the structure volume receiving at least x Gy; V95%Rx and V107%Rx, percentages of the PTV receiving at least 95% and 107% of the prescription dose, respectively. Mean differences were calculated as the first modality minus the second modality. Effect sizes are reported as Cohen's dz for paired t-tests and rank-biserial correlation for Wilcoxon signed-rank tests.* | | | | | |
